# “Tough Things You’re Going to Have to Go Through”: Dyadic Interview Study Including the Perspectives and Needs of Patients and Their Caregivers Post-Hematopoietic Cell Transplant

**DOI:** 10.2196/81971

**Published:** 2026-02-19

**Authors:** Amanda Johnson, Eleanor Smeallie, Chloe Roslin, Michelle Rozwadowski, Evan Shereck, Sung Won Choi

**Affiliations:** 1 Division of Hematology, Oncology, and Bone Marrow Transplant, Department of Pediatrics, University of Utah/Intermountain Primary Children's Hospital, 100 N Mario Capecchi Drive, Salt Lake City, UT, 84113, United States, 1 801-662-4700, 1 801-662-4707; 2 Division of Hematology, Oncology, and Bone Marrow Transplant, Department of Pediatrics, University of Michigan, Ann Arbor, MI, United States; 3 Division of Pediatric Hematology, Oncology, and Bone Marrow Transplant, Department of Pediatrics, Oregon Health & Science University/Doernbecher Children's, Portand, OR, United States

**Keywords:** hematopoietic cell transplant, HCT, bone marrow transplant, BMT, apps, mobile health, caregivers

## Abstract

**Background:**

Patients undergoing hematopoietic cell transplant (HCT) and their caregivers are under a significant amount of stress throughout the HCT process with fear of disease recurrence, graft failure, and many other HCT-related complications. However, the needs and perspectives of patients undergoing HCT and their caregivers as dyadic units over the peri-HCT period are continuing to be studied and are an evolving field of research.

**Objective:**

To better understand patient and caregiver perspectives throughout the HCT course, patients undergoing HCT and their caregivers were able to opt-in to interviews at multiple time points post-HCT as part of a larger study, Roadmap 2.0 (an app intervention trial to support caregivers of patients undergoing HCT).

**Methods:**

Semistructured, dyadic (patient and caregiver) interviews took place around hospital discharge, day +30, +60, +90 and +120 post-HCT. Patient and caregiver discussions at each interview centered around a variety of topics including desired post-HCT information, coping, and additional resources for patients and their caregivers with the goal of gathering feedback to better inform future studies after Roadmap 2.0 and better understand the needs and perspectives of patients undergoing HCT and their caregivers. Interviews were transcribed and double-coded with inductive and deductive content analysis using the framework method to identify key findings.

**Results:**

A total of 10 patient-caregiver dyads participated, resulting in 48 dyadic interviews (1 patient died). Multiple findings emerged out of these rich discussions, including the progression from immediately post-discharge to when patients undergoing HCT and their caregivers were further out from HCT. The progression was as follows: “desire for data and tracking” to “need for specific restrictions and outline on forward progress,” to “need for additional directed information as progressing forward,” to “bigger picture and getting back to life,” and concluding with “reflection and fear.” Most patients and caregivers felt they were provided sufficient general anticipatory guidance throughout the HCT process but called for more specific expectations and guidance on a variety of issues. Many patients and caregivers used multiple coping strategies during HCT, with their coping strategies largely staying consistent over time. Additionally, the need for further acknowledgment and focus on the stress HCT places on caregivers was frequently discussed.

**Conclusions:**

Patients undergoing HCT and their caregivers were largely satisfied with the information and anticipatory guidance they were given but stressed a desire for more specific information throughout their HCT course. A variety of coping strategies are used by patients and their caregivers post-HCT, and these were consistently used over time. However, increased awareness and acknowledgment of the strain HCT places on caregivers are needed within the health care setting and in the general population. Future directions include continued incorporation of qualitative interviews with patients and caregivers as HCT-related interventions and apps.

## Introduction

Hematopoietic cell transplant (HCT) is used to treat both malignant and nonmalignant conditions, using cells from self (autologous) for solid tumors and some lymphomas or donor (allogeneic) for hematologic malignancies and a wide range of other conditions. In both cases, high-dose chemotherapy and/or radiation is used prior to the infusion of hematopoietic stem cells, putting patients at high risk for infections and other complications [1,2]. While HCT and supportive care for HCT complications have greatly improved over the recent decades, there remains a relatively high risk of morbidity and mortality related to HCT [1-4]. Whether receiving an autologous or allogeneic HCT, a full-time caregiver is typically needed for an extended period (up to multiple mo or even longer). Only recently has more public acknowledgment and research been published, acknowledging and focusing on the stress experienced by caregivers of patients undergoing HCT, with caregivers reporting psychosocial distress and diminished quality of life [5-7].

Over recent years, a variety of studies have aimed to help providers and researchers better understand the challenges faced by individuals undergoing HCT and their caregivers as well as determine how best to reach and support them [8-15]. The app, BMT Roadmap, was created by Runaas et al [8] at the University of Michigan over a decade ago with the goal to provide information and support for caregivers of patients undergoing HCT. BMT Roadmap has been developed iteratively, incorporating feedback from rigorous user testing, to create its most recent version, Roadmap 2.0. Roadmap 2.0 incorporates positive, resilience-building activities and was designed for the outpatient setting [9]. In 2020, a mobile randomized controlled trial of Roadmap 2.0 was started (NCT04094844) [16].

**Figure 1.**
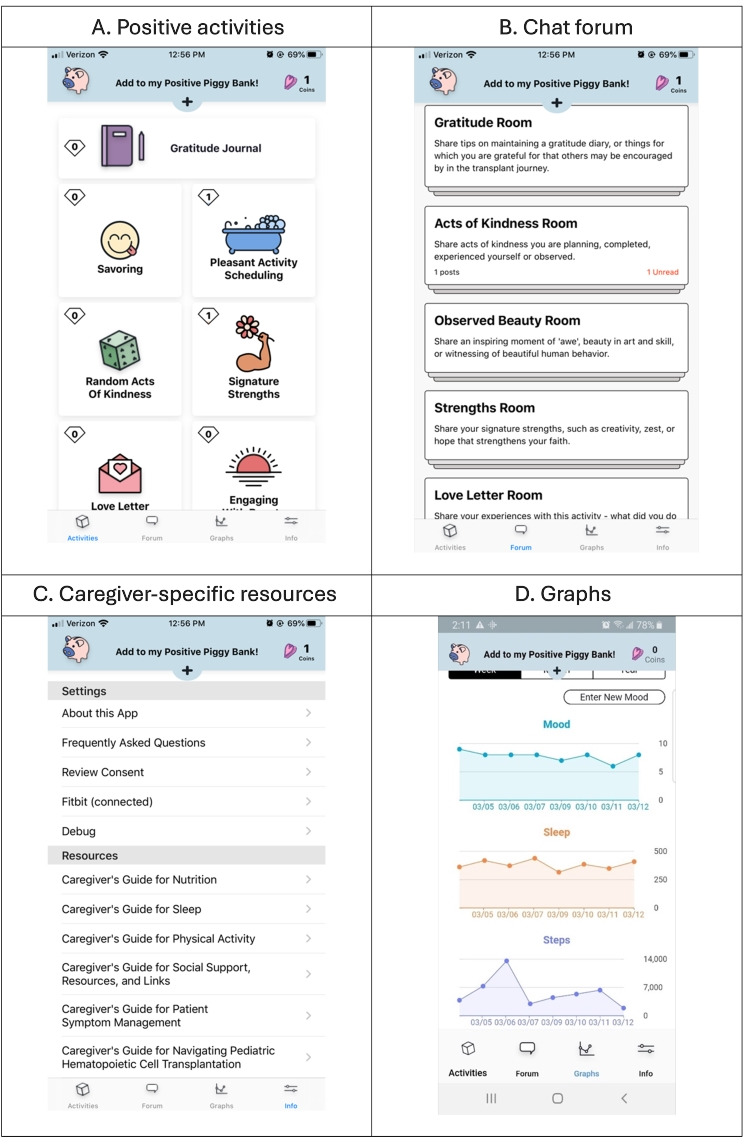
Screenshots from the Roadmap caregiver user interface. (A) Menu of positive activities available in the app. (B) Menu of chat forums available to caregivers. (C) Caregiver resource documents available in the app. (D) Tracking graphs of mood entries as well as sleep and steps imported from the Fitbit. Source: Rozwadowski et al [16], originally published in *JMIR Research Protocols*. This is an open-access article distributed under the terms of the Creative Commons Attribution License (CC BY 4.0).

In the Roadmap 2.0 study, the randomization was at the caregiver level, with an intervention arm of caregivers receiving a menu of positive activities, access to caregiver forums within the app, and a variety of informational guides for caregivers (Figure 1; Multimedia Appendix 1). Caregivers in the control arm received no positive activities, caregiver forums, or informational guides. Caregivers in both arms received a Fitbit that tracked steps and sleep and displayed these results in graphical form in the Roadmap 2.0 app (Figure 1). Caregivers in both arms were asked to record their mood score (scale 0-10) in the Roadmap 2.0 app, which was also displayed in graphical form (Figure 1). The study’s primary endpoint was caregiver quality of life at day +120 as measured by the Patient-Reported Outcomes Measurement Information System Global Health Scale, hypothesizing that caregivers in the intervention arm will have better quality of life when compared to caregivers in the control arm [16]. After 2020, the Roadmap 2.0 study was expanded to include other centers outside of the University of Michigan, extending to Oregon Health & Science University (OHSU) in 2021. The manuscript of the outcomes of the Roadmap 2.0 was recently published, and while there were no significant differences found between the intervention and control groups by Patient-Reported Outcomes Measurement Information System Global Mental or Physical Health Scales, daily mood of caregivers in the intervention arm improved significantly over the course of the study [17].

As an opt-in addition to the Roadmap 2.0 study when OHSU opened as a second center, patients and their caregivers were offered semistructured, dyadic interviews at a variety of time points throughout the study (around hospital discharge, day +30, +60, +90, and +120 post-HCT). The inclusion of this optional portion of the study was to investigate the hypothesis that the support needed for patients and caregivers during the HCT process is both unique to each person and dyad as well as dynamic across the HCT course, which is commonly observed by clinicians throughout the post-HCT course. Additionally, we hoped to gain a better understanding of the support needed by patients and caregivers to further optimize future versions of BMT Roadmap and other mobile health and technology interventions for patients undergoing HCT and their caregivers .

## Methods

### Ethical Considerations

Institutional review board approval was granted at OHSU and oversight approval granted at the University of Michigan, which served as the central or single institutional review board (HUM00176584). The risks and benefits of the study were explained and informed consent was obtained prior to the conduction of optional interviews. All study procedures were conducted in accordance with ethical standards at OHSU and the University of Michigan as well as in accordance with the Declaration of Helsinki. Inclusion criteria for the opt-in interviews were the same as Roadmap 2.0 study inclusion criteria, which included patient age of at least 7 years, caregiver age of at least 18 years, patient and caregiver access to an iPad or cell phone, and patient and caregiver ability to read and understand English. Patient privacy and confidentiality were maintained. Monetary compensation was not provided as part of the study. However, study participants were able to keep their Fitbit following the study.

### Sample and Data Collection

During the consent or assent for Roadmap 2.0, patients and their caregivers were also offered participation in the opt-in interviews. As patients approached discharge, days +30, +60, +90, and +120 post-HCT, patients and/or their caregivers were contacted in person, via email and/or via telephone to arrange semistructured dyadic interviews. Only dyadic interviews were conducted at each of these times with the hope that having both the patient and their caregiver discussing topics together would provide more insight and feedback compared to an individual (patient or caregiver) interview alone. Post-discharge interviews took place within 7 days of discharge. Day +30, +60, and +90 interviews took place up to 2 weeks prior to or as late as 2 weeks following these time points. The day +120 interview took place up to 1 month prior to or as late as 1 month following this time point.

**Figure 2.**
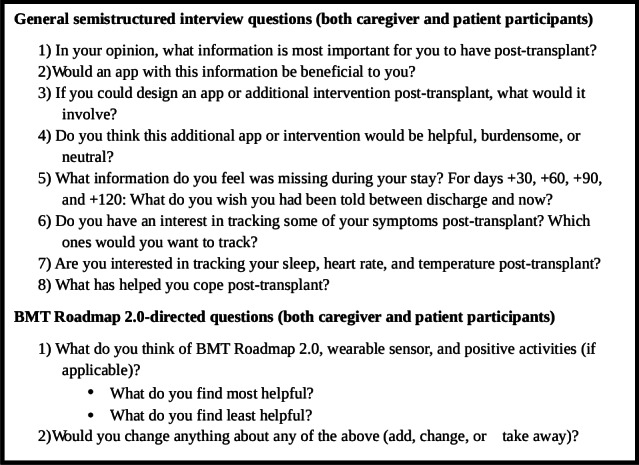
Interview script. BMT: bone marrow transplant.

Interviews took place in person or via telephone, aside from 1 interview where the caregiver was present on video call. AJ completed the interviews with adult patient dyads, and two other research staff members completed the interviews with the adolescent patient dyad given that AJ provided direct care to the adolescent patient. All staff completing interviews were trained in qualitative methods. All interviewers were women, and field notes were taken during the interview for reference following the interview. Interviews were audio-recorded. Interview questions spanned a variety of topics including perspectives on important and missing information during the HCT process, coping, future intervention or app design ideas, and Roadmap 2.0 feedback (Figure 2).

### Data Analysis

Babbletype transcription (Babbletype Inc) was used to transcribe all interviews verbatim. Following transcription, interviews were double-coded by 2 team members (E Smeallie and CR) using NVivo 12.0 (Lumivero). A codebook used in prior BMT Roadmap studies was initially modified to better reflect the interview script [15,16] (Multimedia Appendix 2). Coding by content analysis was both inductive and deductive, using the framework method [18]. As the prior codebook was not organized into themes and the main analytic approach consisted of content analysis, findings were identified rather than the organization of codes into categories and then into themes. Field notes were available for reference but were not directly used for codes. Additionally, the framework method was used to give the research team the ability to analyze the data across the whole data set in addition to grouping by interview time point as well as grouping by dyad [18].

At multiple points during the coding process, team members (E Smeallie and CR) completing coding met with AJ to discuss discrepancies, a consensus was reached when discrepancies occurred, and the codebook was modified as needed. When approximately half of the interviews were completed, a preliminary analysis across all interviews was conducted. Following completion of all interviews, a subsequent analysis was conducted across all interviews, at each time point, and within each dyad over time.

The COREQ (Consolidated Criteria for Reporting Qualitative Research) was used as part of the manuscript preparation (Checklist 1).

## Results

### Interview Results

**Figure 3.**
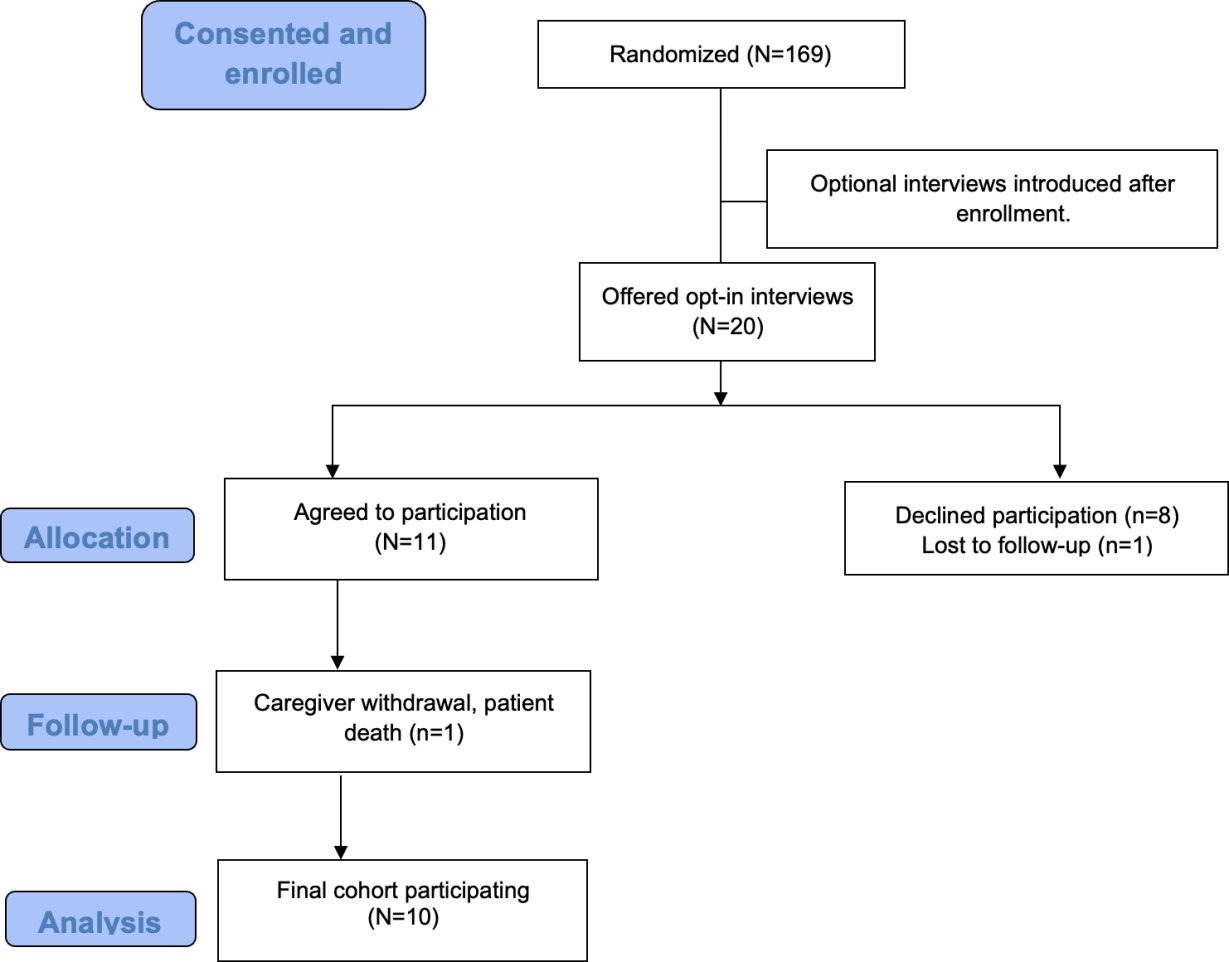
CONSORT flowchart

Interviews took place from November 2021 to July 2022. A total of 20 dyads were offered opt-in interviews. Eight dyads declined, and 1 dyad was lost to follow-up, leaving 11 patient-caregiver dyads who initially opted in for the interview portion of the study. One caregiver withdrew from the study prior to the start of the interviews, and the patient died prior to first hospital discharge, resulting in 10 remaining dyads (Figure 3). Of the 10 dyads, 9 were adult patient-caregiver dyads and 1 was an adolescent patient-caregiver dyad. Among all, 7 dyads were patients and their caregivers from OHSU, and 3 dyads were from the University of Michigan. A total of 7 of the caregivers were part of the positive activities intervention arm. Additional demographics of patients and their caregivers are listed in Table 1.

**Table 1.** Patient and caregiver demographics.

Characteristics	Patients (n=10)	Caregivers (n=10)	Combined (N=20)
Age, median (range)	54.5 (15-73)	58 (42‐68)	56 (15‐73)
Sex, n (%)			
Female	2 (20)	9 (90)	11 (55)
Male	8 (80)	1 (10)	9 (45)
Race, n (%)			
White	9 (90)	9 (90)	18 (90)
Unknown	1 (10)	1 (10)	2 (10)
Ethnicity, n (%)			
Hispanic	1 (10)	0 (0)	1 (5)
Non-Hispanic	9 (90)	10 (100)	19 (95)
Disease, n (%)			
Leukemia	2 (20)	N/A^a^	2 (20)
Lymphoma	1 (10)	N/A	1 (1)
Multiple myeloma	4 (40)	N/A	4 (40)
MDS^b^	2 (20)	N/A	2 (20)
Aplastic anemia	1 (10)	N/A	1 (10)
Caregiver relationship, n (%)			
Parent	N/A	3 (30)	3 (30)
Spouse	N/A	5 (50)	5 (50)
Sibling	N/A	1 (10)	1 (10)
Daughter-in-law	N/A	1 (10)	1 (10)
Intervention or control arm, n (%)			
Intervention arm	N/A	7 (70)	7 (70)
Control arm	N/A	3 (30)	3 (30)

a N/A: not applicable.

b MDS: myelodysplastic syndrome.

Interviews ranged from 10 to 47 minutes (median=21 min). A total of 48 interviews were completed (1 patient died prior to completion of all interviews). Two interviews were unable to be transcribed due to technological reasons, resulting in 46 interviews that were double-coded and analyzed.

While the interview script was consistent throughout the study, overall findings emerged at earlier and later time points looking across all dyadic interviews as they progressed through the post-HCT course. Over time, dyads moved from “desire for data and tracking” and “need for specific restrictions and outline on forward progress” to “need for additional directed information as progressing forward” to “bigger picture and getting back to life” to “reflection and fear.”

**Figure 4.**
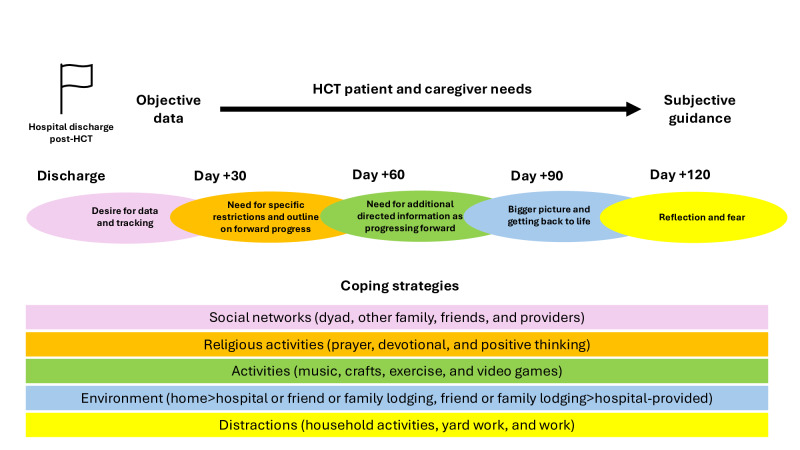
Qualitative interview findings and coping strategies. HCT: hematopoietic cell transplant.

The importance of coping strategies was highlighted with “coping through social networks” being very common among both patients and caregivers. In addition to social networks, multiple other coping strategies were discussed by both patients and their caregivers. The strategies used for patients and their caregivers largely stayed consistent throughout the interviews over time. Interview findings as well as coping strategies are visually represented in Figure 4.

Each finding is described in detail in the following sections with representative quotes within the sections as well as additional quotes in Table 2. In addition to the findings, multiple caregivers highlighted the stress they are under while caring for a patient undergoing HCT, and their experiences are described in further detail after the description of the findings. Finally, a goal of the study was to specifically gather feedback on the Roadmap 2.0 app and study, which is outlined at the end of the results.

**Table 2.** Additional representative quotes for findings.

Finding	Representative quote(s)
Desire for data and tracking	*I am looking at the labs. I’m always looking at his chemistry panel to see what I need to do to boost his whatever he needs to push protein, stuff like that. I think for things going south, I’m probably not just looking at temp. It’s his eyes, his energy, and stuff like that*. [Caregiver, postdischarge] *For me, I’m mostly looking at the vitals. *[Patient, d +30]
Need for specific restrictions and outline on forward progress	*It’s hard because you’re in no man’s land because you’ve never experienced this before. You just are trying to go through that puzzle, figure it all out. Even from now, where is he going to be like in six months from now? What are we looking for long-term? It’s just a thousand things go through your mind that you want to have answers to but nobody really has answers to.* [Caregiver, postdischarge] *What I can and can’t do because right now my legs are so weak but they don’t want me to do anything.* [Patient, d +30]
Need for additional directed information as progressing forward	*I’m thinking more on what they can and can’t eat when they get home...Support on things that he shouldn’t be eating and just like things that he could do.* [Caregiver, d +60] *I’d want to know what you are shooting for in regards to numbers because I didn’t know that those low, low numbers that weren’t even in the normal range got me concerned because of how low – Is something wrong? I’d like to know that that’s okay to be in lower numbers. I guess I just need to have a comfort feeling that – I don’t know how you would do this, but I need to have comfort that everything is okay, this is what you’re looking at, this is what you can do, this is how you progress. I guess an explanation of what to expect*. [Caregiver, d +60]
Bigger pictures and getting back to life	*It’s hard to transition them to live. You’ve got to light a fire under them.* [Caregiver, d +90]
Reflection and fear	*The recovery can occur to me in many different ways…In my case, I have persistent MDS* [myelodysplastic syndrome] *and it seemed to be done in the hospital, but now after the biopsy, it’s still there. It’s not a relapse, it’s not a remission, it’s not a recurrence….It would have been much more helpful had we been aware of the possibilities of what we could be facing.* [Patient, d +120] *I think the biggest thing since day 100 when we left is just not really knowing…what the status of him is. That’s just coming down off of the last three months, I’m sure.* [Caregiver, d +120]

### Desire for Data and Tracking

Earlier in the post-HCT course, patients and their caregivers discussed tracking vital signs and movement via the wearable, recording signs and symptoms, and wanting more data (vitals and laboratories from clinic visits) more often when compared to later time points. Some caregivers also mentioned finding comfort in knowing and tracking data to assess how the patient was doing.

*[L]ike between his last appointment the other day and today’s, one of the numbers went down just a little bit, that would be a great thing to know, because we have three or four days between*.

[Caregiver, postdischarge]

*Yes, just tracking the recovery and like she [caregiver] said, everything is on track and we’re headed in the right direction. That’s the big thing, is just keeping track of how everything is and how well I’m doing and how she’s doing herself.*

[Patient, day +30]

### Need for Specific Restrictions and Outline on Forward Progress

While the majority of patients and caregivers did not feel information was missing in their anticipatory guidance, most patients and caregivers wanted to know specifics on what the patient could or could not do based on their recovering immune system. Additionally, most patients and caregivers called for an explanation from their providers earlier in the post-HCT course on how to move forward and heal rather than the general recommendations they were provided.

*It would be helpful to know what to do going forward, and for how long. Restrictions and what I have to do to keep safe and things like that.*

[Patient, postdischarge]

*[W]hen we asked certain questions of the nurses and that sort of stuff, we got a lot of generalities. It’s understandable generalities. Every patient is different. Part of my recovery in the hospital, some of the caretakers there, ‘Wow, you’re doing far better than most do.’ What could I lag at? Again, something informational...It depends upon the patient, everybody is different, but I’d like a little more information than just that.*

[Patient, day +30]

### Need for Additional Directed Information as Progressing Forward

As the patients and the caregivers progressed through the first weeks and months after discharge, they continued to express a desire for information specific to restrictions and limitations as the patients continued to improve.

*It’s just a lot easier now than it was when we first got released, I’ll say that. It’s a little overwhelming when you first get discharged. At this point we’re home now, and it’s almost back to normal, besides knowing what he can and can’t eat and the meds he’s got to take every day, which is even less than when we got discharged.*

[Caregiver, day +60]

*What can or can’t be done? I still feel like I have a lot of questions about that. What’s appropriate for me to do and not appropriate?*

[Patient, day +60]

### Bigger Picture and Getting Back to Life

While patients and caregivers discussed the desire for a longer-term plan and outlook earlier in the post-HCT course, a strong need for this information came through during the interviews at later points post-HCT. Additionally, patients and caregivers both mentioned the challenges of transitioning out of the immediate post-HCT stage.

*[Y]ou get to this point and you’re starting to think, ‘I’m going to go back to the real world. Can I handle that, or is it really this good? Are things going to be okay?’ It’s just a lot of different feelings that come out that you haven’t really even had to deal with before.*

[Patient, day +60]

*[T]hat’s what I’m looking at. It’s, ‘Here’s how to resume your life.’*

[Patient, day +90]

### Reflection and Fear

Following contemplation of the “bigger picture and getting back to life,” patients and caregivers continued to reflect on what they had just been through with the weight of their underlying diagnosis and fear of rejection, relapse, or other complications despite how far they had come post-HCT.

*I think about the future now...I want to be able to know what to look for if it is coming back.*

[Caregiver, day +120]

*It’s probably been more of the emotional part of it, for me. I don’t know what [he] is experiencing...it’s been a lot of crisis to crisis to crisis. Now it’s like, ‘Oh! There’s no crisis.’ I think dealing with that has been more of a challenge for me. I didn’t know it would be quite that hard...I think that surprised me.*

[Caregiver, day +120]

Another key finding, relevant across time periods, was that many patients and caregivers described social networks as critical to their coping (coping through social networks)*.* Many caregivers also commented on the stress of caregiving for a patient undergoing HCT.

### Coping Through Social Networks

While there was significant variety in coping strategies used by the patients and their caregivers throughout the HCT course, most patients and caregivers had some social network component to their coping. Additionally, most patients and their caregivers used the same or similar coping strategies throughout the study period. One patient, when asked about how he coped post-HCT, answered “my wife,” at nearly every interview. Almost all patients and caregivers mentioned coping through interactions with caregivers, family, friends, or other groups of people who supported them post-HCT.

*At the darkest time, I called my sister when he was in the hospital. It was so bad and I don’t think I was prepared for how bad it was going to be. I just called her and poured my heart out into her. She really helped me.*

[Caregiver, day +30]

*I tell you, where I work, I have a lot of ladies there, golfers, and they’ve been really awesome...I’ve been blessed.*

[Caregiver, day +120]

Both an adolescent and young adult patient mentioned the importance of communicating with their friends via social media and/or gaming platforms throughout their post-HCT course, providing them with a sense of belonging during a time of isolation.

*Talking to people and playing video games.*

[Patient, day +30]

*For me, I think it’s really just been hanging out with my friends online. I actually just checked right now, checked Discord to see if anyone was on. Not yet, it’s early in the day and they’re probably still at work or at school, but just hopping in. There’s a group server that we have, so there’s just a general chat that people will sit in while they play games or do whatever on their computer. You just pop in...Just sit there and talk about whatever.*

[Patient, day +90]

### Challenges of Caregivers of Patients Undergoing HCT

In addition to the above findings, which were common across caregivers and patients, caregivers reiterated the challenges and stress they face when caring for a patient going through HCT. While health care systems and society at large are continuing to work on supporting caregivers, the caregivers in this study provided a call to continue to improve their support, particularly as they face a variety of new stressors during HCT.

*It’s just hard to think sometimes as a caregiver. I just really have been a lot more aware of how it takes – Even to go exercise or do things for yourself at all, when your person that you’re taking care of isn’t at their best or you’re really concerned, it’s just you lose part of your brain too. Energy, willpower, and stuff. That’s been really an eye-opener for me, even though I’ve been a mom for years.*

[Caregiver, day +90]

*I would like something specifically, a program for the caregiver. I get nothing. They don’t even ask me how I’m doing when we go to these appointments. That’s disappointing.*

[Caregiver, day +60]

*[T]here’s a higher level of understanding and resources and outreach and support for a patient than there are for the caregiver. Their [care]givers are expected to be everything...there are not a whole lot of resources on that. Then there’s, I think, an assumption from friends, family, and peers. ‘If you’re a spouse, that’s your job.’ I don’t think there’s an understanding of how it changes [going through HCT].*

[Caregiver, day +90]

### Roadmap 2.0 and General App Feedback

During the interviews, we also asked for structured feedback from both patients and caregivers on Roadmap 2.0 and apps in general. Prior studies by our group and a portion of the larger Roadmap 2.0 study focused on the feedback related to the intervention specifically as a way to continuously improve technology interventions going forward.

Outside of some technical issues, patients and caregivers were overall satisfied with the study and the Roadmap 2.0 app.

*I liked the Gratitude Journal, reminding me to stay positive and grateful for the good things. The Engaging with Beauty, I always think that’s important too, just find the beauty in everything even if you’re at the hospital...Pleasant Activity Scheduling I like, and the Savoring. It’s just all reminders just to do things that are pleasant or positive.*

[Caregiver, day +30]

*I actually like the whole thing. Just to be able to go in, just be able to jot down something in different areas that I was thinking about. I like the Fitbit, it makes sure I’m moving. Overall, I’m pretty satisfied with the whole program-type thing.*

[Caregiver, day +60]

Some patients and caregivers, however, mentioned that they prefer other apps and wearable devices (eg, newer version of Fitbit with additional features). Additionally, some patients and caregivers had coping and intervention strategies for stress that they preferred to use instead of Roadmap 2.0 (eg, reading their Bible or journaling).

*I tried wearing the Fitbit so that it’s pulling in the data but I’m not really an app person. I’m not playing with those Roadmaps or Fitbits or whatever.*

[Patient, day +60]

In addition to specific feedback on Roadmap 2.0, patients and caregivers were asked about app design for the post-HCT setting more generally. The majority of responses centered around an “all-in-one” type of app, where they could access their appointments, test results, and have additional resources (information related to medications, HCT complications, or coping strategies). Many patients and caregivers suggested a more interactive user interface (particularly on the patient-facing app) with exercise plans, activity, and mental health suggestions based on wearable or other input data (eg, heart rate, sleep, and mood), as well as sharing information between the patient and caregiver.

## Discussion

### Principal Findings

In this study, patients actively undergoing HCT and their caregivers engaged in semistructured interviews on their perspectives related to important and missing HCT information, app or intervention design, coping, and provided specific feedback on Roadmap 2.0 as an opt-in portion to the larger Roadmap 2.0 study. Patients and their caregivers showed that they have dynamic needs across the initial post-HCT period, initially focusing on objective data points (ie, laboratories and vital signs) and later transitioning to more long-term subjective guidance. Additionally, the importance of social connections and other coping strategies for both the patient and caregiver was individualized to some extent, but was consistently used throughout the initial post-HCT period. These interviews, as highlighted in the quotes above, led to multiple rich comments and ideas for improving their care broadly as well as specifically, with the hope to apply these findings to future interventions and apps for patient-caregiver dyads, including future versions of the BMT Roadmap app.

The next version of the Roadmap app is in its planning phase with the goal to make the app interface more interactive with a “just-in-time” approach where a patient or caregiver will receive additional prompts or suggestions after entering data. For example, a caregiver rating their mood 2 on a scale of 10 within the app may receive a positive message of encouragement or be provided additional resources after entering their low mood score. Additionally, our group has been exploring additional interventions to support caregivers during the peri-HCT period with multiple caregiver support projects underway, including specific work on how to capitalize on a caregiver’s social network for support with needs and tasks [19]. While implementing the rich feedback gathered in this study promptly will help support some patients and their caregivers, we need additional studies to gather further patient and caregiver perspectives to improve the reach of the app.

As patients and their caregivers transition from the hospital to the outpatient environment, where vital signs and labs are checked much less frequently, participants’ “desire for data and tracking” resonating in earlier time points post-HCT is not surprising. Similarly, participants’ “need for specific restrictions and outline on forward progress” is also fitting for earlier in the post-HCT course when one of the primary goals is to discharge from the hospital and it is impossible to give directed guidance on every potential exposure situation the patient and their caregiver are going to face outside of the hospital. Additionally, about 12% (3318/28,356) of adult autologous transplant patients and 24% (4201/17,213) of adult allogeneic transplant patients experience unplanned readmission to the hospital within 30 days of discharge for a variety of complications that span from minor, short hospital stays to life-threatening illnesses, making outlines of the future difficult to predict early in the post-HCT course [20].

As patients and caregivers spend more time outside of the hospital after discharge, additional anticipatory guidance is certainly needed in keeping with participants’ “need for additional directed information as progressing forward.” During the second and third months after HCT, patients are likely beginning to regain strength and overall feel better, making it more difficult to remember that their immune system is still immature and they are at continued high risk for infections.

Between 3 and 4 months after HCT, patients and their caregivers are typically allowed to return home if they needed temporary local housing due to their home being outside of the immediate area of the HCT center and their appointments are spaced out further. Just prior to and around this time, patients and their caregivers begin wondering what returning home and the follow-up plan will look like, and thus a desire for the “bigger picture and getting back to life,” which is commonly observed clinically and confirmed in this study. Additionally, similar to the anxiety experienced around the time of discharge, “reflection and fear” occur just prior to and just after day +100 and were discussed by multiple patients and caregivers during the day +120 interview.

Finally, the stress HCT places on patients and their caregivers was significant, and coping through a variety of mechanisms unique to each patient and their caregivers was observed. It is not surprising that similar coping strategies were used throughout the HCT process given that many patients and caregivers experience a prior diagnosis (malignant or nonmalignant) that later leads to a recommendation for HCT. Thus, their coping skills were tested prior to proceeding to HCT. Despite having previously tested coping strategies, many caregivers drew attention to the challenges of caregiving for a patient undergoing HCT [1]. Additional quantitative, qualitative, and mixed methods studies are needed as we gain further insight into these stressors and attempt to implement strategies to improve the burden of caregivers of patients undergoing HCT.

### Strengths and Limitations

This study has multiple strengths including a geographically diverse sample and the use of repeated interviews over time, which allowed for building trust and a deep understanding of interviewees’ experiences. Multiple dyadic interviews were conducted at a variety of time points, allowing for triangulation and increased trustworthiness of the data to enhance both the validity and reliability of our data. Additionally, having the majority of interviews conducted by AJ allowed for increased consistency between interviews. Coding of the data by more than one researcher outside of the interviewers brought additional perspectives and provided reflexivity which guarded against bias in the interpretation. In terms of limitations, these interviews were optional and thus were more likely to involve individuals with more polarized views (either very positive or very negative). The interviews were limited in numbers compared to the larger study, which was a result of OHSU staff entering the study later and conducting the interviews. However, based on general qualitative study guidance, we believe our sample size was sufficient to achieve saturation of findings. Additionally, the majority of caregivers who opted in to these interviews were on the intervention arm of the Roadmap trial, which may have influenced the results (ie, they had additional positivity resources and could be more likely to have positive views and responses to questions). As the interview script consisted of semistructured questions, responses may certainly be more limited in comparison to fully open-ended questions. While overall the dyadic interview method is felt to be a strength in terms of allowing the patient and caregiver to engage in a richer discussion together, it is possible that either the patient or the caregiver was not fully comfortable sharing some responses in front of the other during these interviews. It was also notable that the majority of caregivers were White females of White male adolescent and adult patients, limiting the generalizability of their experiences. It was a goal amongst our group to reach more diverse populations of patients and caregivers in our smaller studies to better inform our larger upcoming studies.

### Conclusions

The field of mobile health and technology continues to expand, and apps are rapidly being developed. Health apps make up a smaller niche and are increasingly being used. Integration of these apps with the electronic health record is beginning to be done as well [21,22]. With this in mind, engaging end users (eg, patients, caregivers, and providers) in the design process of these apps is extremely important for usability as well as improving these interventions. This study shows the strengths of mixed methods design for intervention design and improvement, highlighting where patients undergoing HCT and their caregivers need further information, guidance, and support during the HCT process.

Results of the larger Roadmap 2.0 study have recently been published, focusing primarily on quantitative findings [17]. Based on our prior work and results of this study, we plan to continue to incorporate qualitative interviews in future Roadmap studies to further understand the patient and caregiver experience as well as determine additional patient and caregiver needs and resources post-HCT. Prior to the next Roadmap study, our group is conducting a variety of smaller studies to optimize the next Roadmap platform and other patients undergoing HCT and caregiver interventions going forward.

While these studies are still underway and our understanding and recommendations will continue to mature, we believe the results of this study offer general suggestions to health care providers and social support networks of patients undergoing HCT and their caregivers. First, as patients undergoing HCT and their caregivers progress through the post-HCT trajectory, providing specific advice where possible is appreciated, whether based on the provider’s recommendation or institutional practices, such as outlining and reviewing dietary and activity restrictions at regular intervals. While general advice is appreciated, subjective guidance specific to each patient and caregiver situation is also appreciated. Finally, acknowledgment of and support for caregivers of patients undergoing HCT during the peri-HCT period is needed. Potential suggestions could include having a member of the care team check in with the caregiver at clinic visits in addition to improving respite programs for caregivers of patients undergoing HCT to allow them time for their own health (eg, time to exercise, nap, or run errands without fear of leaving the patient alone).

## Supplementary material

10.2196/81971 Multimedia Appendix 1 Roadmap 2.0 app multicomponent featured defined.

10.2196/81971 Multimedia Appendix 2 Codebook.

10.2196/81971 Checklist 1 COREQ checklist.

## Abbreviations

COREQ: Consolidated Criteria for Reporting Qualitative Research
HCT: hematopoietic cell transplant
OHSU: Oregon Health & Science University
