# Supplementary material for: “Tough Things You’re Going to Have to Go Through”: Dyadic Interview Study Including the Perspectives and Needs of Patients and Their Caregivers Post-Hematopoietic Cell Transplant
Source: J Particip Med. 2026 Feb 19;18:e81971. doi: 10.2196/81971 (PMC12919964; doi:10.2196/81971)
Supplement: Multimedia Appendix 2 [file jopm-v18-e81971-s002.docx]

**Multimedia Appendix 2:** Codebook.

| **Code** | **Definition** |
| --- | --- |
| INFO-OTHER, INFO-CAL, INFO-VIT | CG/PT describes the information they find most important to have post-transplant |
| Y-APP | App that helps track this INFO would be helpful |
| N-APP | App that helps track this INFO would *not* be helpful |
| DES-RM, DES-GEN | CG/PT mentions what they would want to involve in their post-transplant app |
| HELP | CG/PT mentions that an app with this information would be helpful post-transplant |
| BURD | CG/PT mentions that an app with this information would be burdensome post-transplant |
| NEU | CG/PT mentions that an app with this information would be neutral post-transplant |
| MIS-SCHED; MIS-MED, MIS-OTHER (if doesn't fit in those categories) | CG/PT says what they wished they had known between discharge & now (only applicable to day +30, day +60, etc.) |
| COMPL | CG/PT says hospital told them all they needed to know/completely understood post-discharge goals |
| Y-TRACKSX | CG/PT says they are interested in tracking symptoms post-transplant |
| N-TRACKSX | CG/PT says they are *not* interested in tracking symptoms post-transplant |
| Y-TRACKVIT | CG/PT says they are interested in tracking heart rate, sleep, temperature, etc. post-transplant |
| N-TRACKVIT | CG/PT says they are *not* interested in tracking heart rate, sleep, temperature, etc. post-transplant |
| TEMP | CG/PT mentions that an app tracking temperature would be helpful |
| MHELP-FB; MHELP-RM, MHELP-PA | CG/PT mentions most helpful part of BMT Roadmap |
| LHELP-FB, LHELP-RM, LHELP-PA | CG/PT mentions least helpful part of BMT Roadmap |
| COPE-FAM, COPE-DYAD, COPE-SOCIAL, COPE-EXERCISE, COPE-OTHER (if doesn’t fit in those categories) | CG/PT explains what helped them cope post-transplant |
| LOC-HOSP | CG/PT explains being in the hospital impacted ability to participate in the study/complete positive activities |
| SUGG | CG/PT makes suggestion (pos/neg) on general study suggestions that would be helpful to include in recruitment/onboarding process |
| SUGG-A | CG/PT makes suggestion (pos/neg) on features that would be helpful to include in the app |
| POS | CG/PT mentions something positive about the study/ apps [asked/coded separately from MHELP] |
| NEG | CG/PT mentions something they do not like about the study/apps |
| GEM | Coder discretion - sees an amazing quote/“gem” |
| TECH | CG/PT mentions technical issues |

CG=caregiver, PT=patient
